# Multiple Myeloma Involving Skin and Pulmonary Parenchyma after Autologous Stem Cell Transplantation

**DOI:** 10.1186/1756-8722-2-48

**Published:** 2009-11-13

**Authors:** Yuan Yuan, Rosemary Wieczorek, David L Green, Perry Cook, Harold Ballard, David J Araten

**Affiliations:** 1 Division of Hematology, New York Veteran's Hospital, New York, USA; 2 Department of Pathology, New York Veteran's Hospital, New York, USA; 3 Division of Hematology, New York University School of Medicine and the NYU Langone Clinical Cancer Center, New York, USA; 4 Current Address: Division of Oncology/Hematology, Loma Linda University Cancer Center, 11234 Anderson Street, A600 Loma Linda, CA 92354, USA

## Abstract

Pulmonary involvement and skin involvement are rare complications of plasma cell neoplasms. Here we describe what may be the first reported case of a patient with relapse in both of these sites following autologous peripheral blood stem cell transplantation.

## Text

We report a 58-year-old man with diffuse pulmonary parenchymal plasma cell infiltration and skin nodules. Five years previously he had been treated successfully with VAD (vincristine, adriamycin and dexamethasone) chemotherapy and an autologous peripheral blood stem cell transplant for multiple myeloma. Four years later he relapsed with anemia, hypercalcemia, and an IgG of 6170 mg/dl. Skeletal survey showed no lytic lesions, the marrow was 30% infiltrated by plasma cells with a t(11;14)(q13;q32) and a del 13q14.3 abnormality. The paraprotein level transiently responded to thalidomide-dexamethasone, but he in turn developed hyperammonemia, which responded to the addition of bortezomib and liposomal pegylated doxorubicin to the regimen.

**Figure 1:**
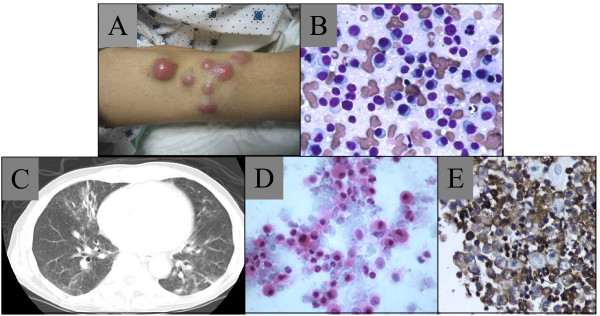
**(A) Skin nodules; (B) Wright Giemsa stain, aspirate of skin nodules; (C) Chest CT showing diffuse infiltrates; (D) H&E of BAL; (E) Kappa staining of BAL**.

During the 3^rd ^cycle of this regimen, subcutaneous nodules appeared on all four extremities, and a needle aspirate revealed plasma cells (figure 1A, B). The WBC rose to > 40,000/cmm with 50% circulating plasma cells. He developed dyspnea and cough, and CT revealed peri-bronchial thickening and patchy opacities diffusely (figure 1C). Bronchoalveolar lavage (BAL) demonstrated kappa restricted plasma cells (Figure 1D, E). He soon after expired, concurrent with a rise in the ammonia level.

## Discussion

In plasma cell neoplasms, pulmonary complications are common, but infection is the most common cause. In a series of 958 patients with myeloma, 10% had pulmonary findings, but in only 1 case was plasma cell involvement demonstrated histologically, and the clinical course was suggestive of plasma cell involvement in only 3 other cases [1]. In the literature, there are additional cases of pulmonary involvement: some of these had advanced or treatment-refractory disease, like our patient [2-4], whereas in several cases, the lung was the initial site of presentation [5-8]. Likewise, skin involvement is rare, but this too has been reported [9,10]. We believe that this is the first case patient reported to have both sites of involvement as a complication of relapsed disease after autologous stem cell transplantation. The presence of circulating plasma cells and hyperammonemia are both known to be indicators of advanced disease. Plasma cell neoplasm should be kept in the differential diagnosis of any pulmonary infiltrate not responding to antibiotics or as a possible cause of skin nodules in a patient with a history of myeloma, plasmacytoma, or MGUS.

## Competing interests

The authors declare that they have no competing interests.

## Authors' contributions

All authors were involved in the provision of clinical care of the patient and the collection of data and the review of the manuscript. RW performed immunohistochemical analysis. YY drafted the manuscript and reviewed the literature, as did DA, who performed the final edits.

## Consent

The Institutional IRB approved the submission of this manuscript, which was considered by the editors to be a proxy for the patient's informed consent for the publication of the manuscript and accompanying images, in light of the fact that the patient had expired. A copy of the written documentation of this is available for review by the Editor-in-Chief of this journal.
